# Banat donkey, a neglected donkey breed from the central Balkans (Serbia)

**DOI:** 10.7717/peerj.8598

**Published:** 2020-03-03

**Authors:** Ljubodrag Stanisic, Jelena M. Aleksić, Vladimir Dimitrijevic, Branislav Kovačević, Jevrosima Stevanovic, Zoran Stanimirovic

**Affiliations:** 1 Department of Reproduction, Fertility and Artificial Insemination, Faculty of Veterinary Medicine, University of Belgrade, Belgrade, Serbia; 2 Institute of Molecular Genetics and Genetic Engineering (IMGGE), University of Belgrade, Belgrade, Serbia; 3 Department of Animal Breeding and Genetics, Faculty of Veterinary Medicine, University of Belgrade, Belgrade, Serbia; 4 Institute of Lowland Forestry and Environment (ILFE), University of Novi Sad, Novi Sad, Serbia; 5 Department of Biology, Faculty of Veterinary Medicine, University of Belgrade, Belgrade, Serbia

**Keywords:** *Equus asinus*, Donkey breeds, The Balkans, Conservation of genetic resources, Genetic diversity, Mitochondrial genome, Morphological traits, Nuclear microsatellites

## Abstract

The dominant donkey breed in the Balkans is the mid-sized Balkan donkey with a grey to chocolate coat color. Local breeders from Serbia, however, still maintain a few larger individuals of a lighter coat color, named Banat donkey, and speculate that they are descendants of a Spanish donkey heard that had been transferred to the Banat region by the Hapsburg Queen Maria Theresa in the XVIII century for a specific purpose, to work in local vineyards. We have previously found a unique nuclear gene-pool and a prevalence of mitochondrial Clade 2 haplotypes in several such animals. In this study, we: (i) perform a comparative analysis of 18 morphological traits of the Banat donkey (seven individuals), Balkan donkey (53 individuals from two sub-populations of this breed) and the potential hybrids (eight individuals), and demonstrate the morphological distinctiveness of the Banat donkey, highlighting the diagnostic traits for distinguishing the breed: hip height, croup width, body length and chest depth; (ii) re-analyse published nuclear microsatellite data for these groups, and reveal that, although severely depopulated, the genetically distinct Banat donkey is not severely affected by the loss of genetic diversity and inbreeding; (iii) demonstrate that previously published Banat donkey mitochondrial haplotypes, analyzed genealogically together with those reported in ancient and modern individuals from Spain, Italy, Turkey, Cyprus and Africa, are shared with three Spanish breeds and individuals belonging to Amiata and some other Italian breeds. A unique morphological feature present in Banat and Somali wild donkeys, but also in Amiata donkeys, black stripes on legs, suggests that the origin of Clade 2 donkeys may be much more complex than previously thought. Actions to preserve the Banat donkey, a valuable but critically endangered genetic resource (<100 individuals), are urgent.

## Introduction

The domestication of donkey, *Equus asinus* L., Equidae, in the arid regions of north-eastern Africa dates back to ∼7,000 years ago (Beja-Pereira et al., 2004; Marshall, 2007; Rossel et al., 2008; Rosenbom et al., 2015). Molecular evidence supports two independent domestication events because two distinct lineages, Clade 1 and Clade 2, have been observed based on the variability of maternally inherited mitochondrial DNA (mtDNA) (Beja-Pereira et al., 2004; Kimura et al., 2011). Molecular data also highlighted ancient Nubian wild donkey as an ancestor of Clade 1 donkeys (Beja-Pereira et al., 2004; Kimura et al., 2011), while the ancestors of Clade 2 donkeys are still unknown because the findings that they trace their origin to a relative of the Somali wild donkeys (*Equus africanus somaliensis*, Noack, 1884), which is probably already extinct (Beja-Pereira et al., 2004; Kimura et al., 2011), have been questioned recently by several authors (e.g., Kefena et al., 2014; Rosenbom et al., 2015; Stanisic et al., 2017; Xia et al., 2019).

Donkeys were introduced into the Mediterranean Basin and the Balkans by Greeks, who brought them from their African colonies in the 2nd millennium BC (Vilá, Leonard & Beja-Pereira, 2006). Stanisic et al. (2017) demonstrated recently that, at least in the case of the Balkan Peninsula, these introductions proceeded in multiple waves, because donkeys belonging to Clade 2 appeared in Greece prior to those belonging to Clade 1, however, they diversified and expanded throughout the Balkans and Europe later than Clade 1 donkeys. The donkey breed present today in the Balkan Peninsula is the Balkan donkey, usually regarded as unselected, unstructured and traditionally managed donkey breed (Kugler, Grunenfelder & Broxham, 2008). However, according to Stanisic et al. (2017), the history and the current genetic structure of the endangered and depopulated donkey population in the Balkans was much more complex than previously reported (Pérez-Pardal et al., 2014). Furthermore, genetically distinct sub-populations of the Balkan donkey, as well as new breeds that may be acknowledged (e.g., Ivankovic et al., 2002) or those that are neglected, still uncharacterized and brought to the brink of extinction, may be present in this region (Stanisic et al., 2017).

The donkey population from the Iberian Peninsula (Spain) also derives from two ancestral sources (e.g., Aranguren-Mendez et al., 2004), and, according to Epstein (1984), there has been occasional direct transfer of animals from Africa via the Strait of Gibraltar in the past. Furthermore, the extant maternal landscape of the Spanish Catalana, Mallorqina and Zamorano-Leonessa breeds is highly similar to that which can be observed in the two African donkey populations, one from Morocco and the other from Zimbabwe (Aranguren-Mendez et al., 2004). On the other hand, Spanish donkey breeds, such as for instance the Catalonian donkey, have been commonly used in the past few centuries to improve not only European but also American breeds (Jordana & Folch, 1996; Jordana et al., 2016). To the authors’ best knowledge, the genetic impact of Spanish donkeys on those from the Balkan Peninsula has not been recorded, but it is possible, since, according to local breeders from Serbia, in the XVIII century the Habsburg queen Maria Theresa requested that a heard of Spanish donkeys be transferred to the north-eastern part of Serbia, the Banat region, to work in local vineyards. These donkeys were stronger, taller and thinner than the local Balkan donkeys, and were thus able to pass more easily between the rows of grapevine. Such a specific use of introduced Spanish donkeys would actually imply that their original traits would have to be maintained in the new environment over time. This would suggest also that these animals, named by local people the Banat donkey, may be characterized by mtDNA profiles similar to those found in Spanish donkeys.

The Banat donkey differs morphologically from the Balkan donkey not only by its larger body, but also by different coat colour and unique pigmentation scheme, with a cross on the back and black stripes on legs resembling those typical for Somali wild donkeys (Groves, 1986; Moehlman, 2002) present today in Somalia, Ethiopia and Eritrea (Moehlman, 2002). Such pigmentation represents primitive markings of the species (Johnson & Johnson, 2008) typical also for the Italian Amiata donkey (Sargentini et al., 2009; Sargentini et al., 2018). According to Stanisic et al. (2017), the Banat donkey is also characterized by a unique nuclear gene pool, as inferred from the analysis with 11 nuclear microsatellites in both Banat and Balkan donkeys, and furthermore, it predominantly harbours Clade 2 mtDNA haplotypes that are nowadays less abundant in Ethiopian donkeys (Kefena et al., 2014), and more common in those from south-west Asia and Europe, including several Spanish (Aranguren-Mendez et al., 2004) and Italian donkey breeds (Cozzi et al., 2017). However, despite these numerous indications of morphological and genetic distinctiveness, the nowadays neglected and severely depopulated Banat donkey is not yet formally acknowledged as a distinct donkey breed.

Donkeys are still amongst the least studied and most neglected livestock species of the world (Blench, 2000). The persistence of donkey breeds worldwide is threatened by the rapid decline of their populations (e.g., Ivankovic et al., 2002; Ciampolini et al., 2007; Bordonaro et al., 2012; Quaresma et al., 2014), and actions concerning their conservation and establishment of breeding programs are therefore urgently needed (FAO, 2015). They depend on proper identification of donkey breeds, data on breeding populations and their phenotypic and genetic characterization which are still incomplete (Kugler, Grunenfelder & Broxham, 2008). The aim of this study is to shed more light on donkey breeds from the Balkans by assessing whether the neglected and uncharacterized Banat donkey may be acknowledged as a distinct donkey breed in Serbia, following the definitions for donkey breeds recognition given by Alderson (2003), and whether speculations on the Spanish origin of these donkeys are supported. To this end, we: (i) carry out a comparative analysis of 18 morphological traits of the Banat donkey (seven individuals), Balkan donkey (53 individuals from two sub-populations of this breed, previously studied by Stanisic et al., 2015) and possible hybrids between Banat and Balkan donkey (eight individuals); (ii) take advantage of the dataset of Stanisic et al. (2017) with nuclear microsatellite genetic profiles to compare these groups at the genetic level; (iii) use available mtDNA haplotypes found in Banat and Balkan donkeys (Stanisic et al., 2017), together with those reported in ancient and modern individuals from Spain, Italy, Turkey, Cyprus and Africa, to assess their genealogical relationship.

## Materials & Methods

### Banat donkey population in Serbia, sample size and data collection

According to the Domestic Animal Diversity Information System (DAD-IS) of the FAO (accessed May 25, 2019), the estimated number of donkeys in Serbia is 500–1,000. A small number of adult donkeys and foals is kept in individual households usually in rural regions, while the number of sexually mature individuals registered in 2015 at three localities where large donkey herds are currently maintained, Special Nature Reserve “Zasavica” (ZA), Stara Planina mountain (SP), and in vicinity of the Kovilj village (KO), is 281. These animals are classified to the endangered Balkan donkey breed found in the Balkan Peninsula (Kugler, Grunenfelder & Broxham, 2008; Stanisic et al., 2015; Stanisic et al., 2017). In spring 2017, we visited these localities and recorded all individuals that differ morphologically from the Balkan donkey and, according to local breeders, belong to the Banat donkey.

**Figure 1:**
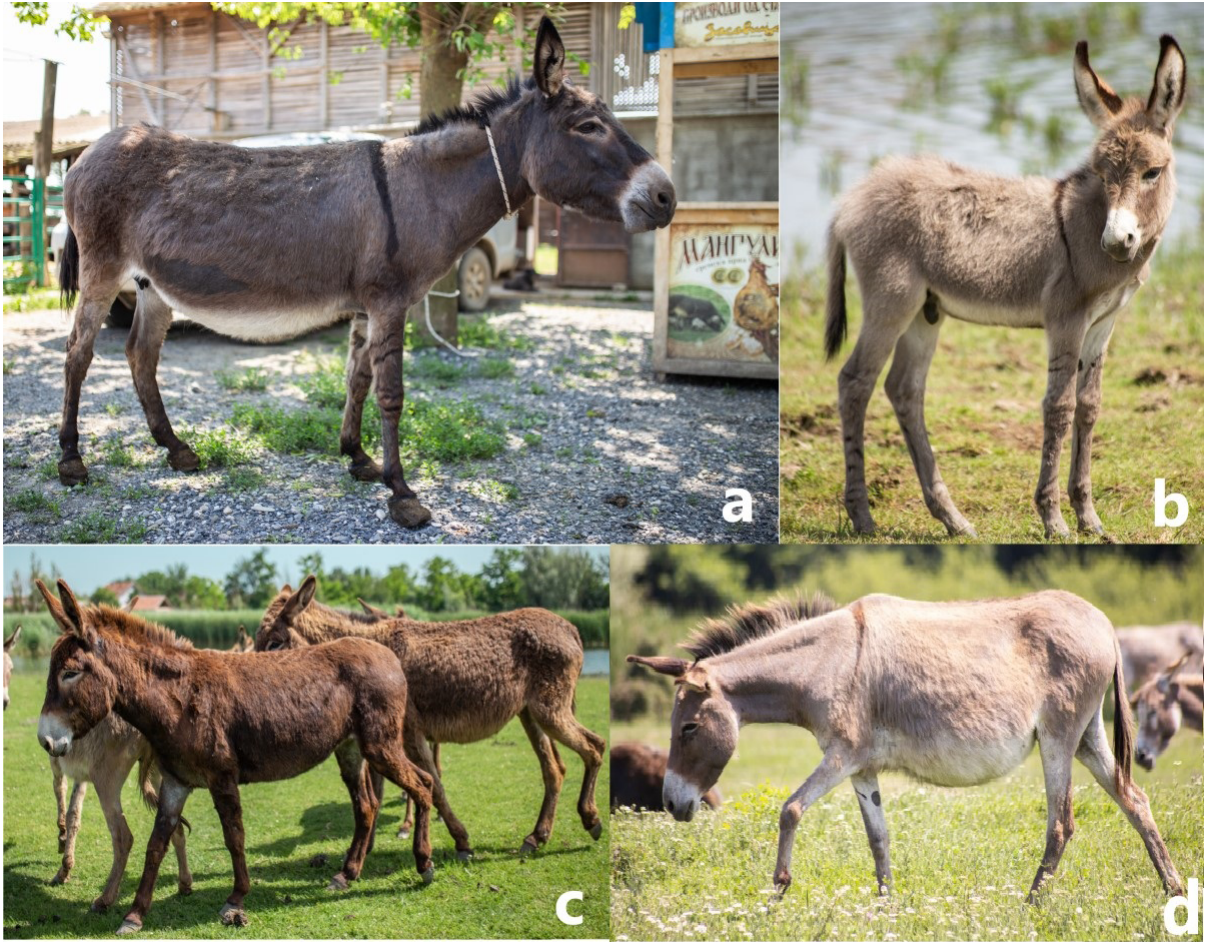
Photos of the Banat donkey female (A) and foal (B), a potential hybrid between Banat and Balkan donkey (C), and Balkan donkey (D). Photo credit: I. Stanivuković.

Since the controlled mating between Banat donkeys over the past 25 years was more or less common only in ZA, we used animals from this locality in our study. These individuals have already been studied genetically by Stanisic et al. (2017), and were characterized by the unique nuclear gene pool and prevalence of the mtDNA Clade 2 haplotypes. However, after re-capturing them, we observed that only eight out of 16 animals were indeed morphologically different from the Balkan donkey (Figs. 1A and 1B). The remaining ones, although assigned to the Banat donkey based on their nuclear genetic profiles, were visually indistinguishable from the Balkan donkey because they were mid-sized animals, with grey to chocolate coat color, dark cross on shoulder and silver bright coat pigmentation on the chest and abdomen, along the medial side of the extremities and around the nose and the eyes (Fig. 1C). Thus, these animals were delineated as potential hybrids (HY) and were separated from eight individuals representing Banat donkeys (BanD). All but one BanD individual were adult females, three to eight years old (average age 5.25 years). The average age of HY, which were all females, was 5.75 years.

Eighteen morphological traits described by Stanisic et al. (2015) were measured in animals used in the present study (Fig. S1) approved by the Ethical Committee of the Faculty of Veterinary Medicine, University of Belgrade, Serbia (approval reference number 01-19/7). All re-captured individuals were carefully handled by trained laborers, positioned on flat and hard grounds with parallel legs, and calmed before the non-aggressive measurement was carried out. A single person recorded all the measurements taken from the right side of the body following the procedure used previously by Stanisic et al. (2015) for assessing body measurements in the Balkan donkey. That is, six body variables (carpal circumference, chest circumference, ear length, head length, tarsal circumference and tibia circumference) were measured with a measuring tape, and 11 (back height, body length, carpal height, chest depth, chest width, croup length, croup width, head width, hip height, tarsal height and wither height) with the Lydtin stick. The body weight (BW) of each animal was calculated following the formula given by Pejić (1996): BW (kg) = [(chest circumference)^2^ × (body length)]/11877.

We analysed morphological traits of BanD and HY together with those available for 53 female Balkan donkeys from Serbia whose morphological features have been studied previously by Stanisic et al. (2015). These animals were studied genetically as well (Stanisic et al., 2017), and were selected among 77 individuals based on the assignment probability (*q* ≥ 0.60) to one of the two nuclear gene pools obtained in the Bayesian clustering analysis performed with STRUCTURE v. 2.3 (Pritchard, Stephens & Donnelly, 2000). Two gene pools in question, so-called blue gene pool (BGP) and red gene pool (RGP), represent two sub-populations of the heterogeneous Balkan donkey breed (Stanisic et al., 2017). We therefore assembled two groups using selected individuals, BalkD-BGP (average age 5.27 years) and BalkD-RGP (average age 5.03 years), regardless on the origin of individuals (ZA, SP and KO). In that way, we used 26 females belonging to the BGP (14 from ZA, nine from SP, and three from KO), and 27 females belonging to the RGP (five from ZA, five from SP and 17 from KO).

### Statistical analyses

For each of the four assembled donkey groups (BanD, HY, BalkD-BGP and BalkD-RGP), mean, median and quartile range of each morphological variable (expressed in cm or kg) as well as coefficient of variation (CV, expressed in %) were assessed with STATISTICA 13 (TIBCO, Palo Alto, CA, USA).

Multivariate analysis of variance (MANOVA) was used to test the impact of differences between studied groups on variation of morphological parameters. The significance of tests was assessed with both Hotelling-Lawley and Wilks approximate *F*-tests. Hotelling’s T2 test was used for testing the significance of differences between donkey groups based on all measured parameters. Then, one-way Analysis of Variance (ANOVA) was applied to test the null hypotheses about influence of differences between groups on variation of each character. Statistical significance of differences between groups based on ANOVA was assessed using the Tukey’s test (HSD-test). These analyses were performed with R programming language (R Core Team, 2017), and ‘Hotelling’ package (Curran, 2018) implemented in R for Hotelling’s T2 test.

To further describe the importance of the influence of differences between donkey groups on variability on studied parameters, we calculated contribution of variances to the total expected variances (intraclass correlation coefficient). Expected variances were calculated using STATISTICA 13. The same software was used for Principal component analysis (PCA) performed in order to group measured parameters according to their loadings (i.e., correlation coefficients) with selected and rotated principal components (PC). The most informative PCs were selected according to the Kaiser’s role (*λ* > 1, where *λ* stands for eigenvalue of particular PC), and then rotated by the Varimax method to maximize variance of loadings between measured parameters and PCs within PCs. Since PCs are in orthogonal position against each other, the correlation between them is 0, and thus, the measured parameters that have their highest loadings with the same PC were considered to be correlated and to belong to the same PCA-group, with weak correlations with parameters from other PCA-groups. Relationships between studied groups were assessed in cluster analysis by calculating a matrix of squared Mahalanobis distances between groups used to build a dendrogram with unweighted pair-group method with arithmetic mean (UPGMA), and by Canonical Discriminant Analysis (CDA). Also, Stepwise Forward Discriminant Analysis was used to evaluate the importance of studied morphological parameters (predictors) for discrimination of examined donkey groups. For each successive discriminative model, the percentage of correct allocation is calculated. Importance of parameters was assessed according to order by which parameters entered the discriminative model and the difference in the percentage of correct allocation between the model with and the model without included parameter. These analyses were performed with STATISTICA 13.

Genetic profiles of individuals belonging to four groups (BanD, HY, BalkD-BGP and BalkD-RGP), assessed previously by Stanisic et al. (2017) with 11 microsatellites (AHT4, AHT5, ASB23, CA425, HMS2, HMS3, HMS6, HTG6, HTG7, HTG10 and VHL20), were used for the assessment of the standard genetic diversity parameters, i.e., number of different alleles (*A*), effective number of alleles [*Ae* = 1∕(1 − *H*_*E*_)], number of private alleles (*PA*), observed heterozygosity (*H*_*O*_), expected heterozygosity (*H*_*E*_), and inbreeding coefficients (*F*_*IS*_) in each group, with GenAlEx 6.5 (Peakall & Smouse, 2012). The same software was employed for Principal Coordinates Analysis (PCoA) used for assessing genetic affinities of groups, and for estimating pairwise group *F*_*ST*_ values and the number of migrants per generation (*Nm*) calculated as [*Nm* = 1∕4(1∕*F*_*ST*_ − 1)]. The matrix of pairwise group *F*_*ST*_ values was summarized by two-dimensional scaling (MDS) using Paleontological Statistics (PAST) ver. 3.0 (Hammer, Harper & Ryan, 2001). The analysis of molecular variance (AMOVA, Excoffier, Smouse & Quattro, 1992), aimed at partitioning the overall molecular variation to within- and among-group variation, was performed with Arlequin ver. 3.0 (Excoffier, Laval & Schneider, 2005). Bayesian clustering analysis was carried out with STRUCTURE in order to assess the optimal number of independent genetic groups (*K*). Burn-in length and run length were 500,000 iterations each, and ten independent runs for each of the assumed *K* = 1–6 were performed under the admixture and correlated allele frequencies models. The optimal *K* value was determined by the highest mean estimated log probability of data (Pritchard, Stephens & Donnelly, 2000).

Based on the variability of the mtDNA D-loop, Stanisic et al. (2017) reported 19 mtDNA haplotypes in Banat and Balkan donkeys (h1–h9 belonging to the Clade 1, h10–h19 to Clade 2, GenBank accession numbers KR081377 –KR081395) of which h12 was predominant in the Banat donkey that harboured also a rare h18. These haplotypes were used together with those found in six Spanish breeds and two populations from Africa (Morocco and Zimbabwe) (GenBank accession numbers AF416593, AF416594, AF416595, AF416596, AF416597, AF416598, AF416599, Aranguren-Mendez et al., 2004), six Italian donkey breeds (KX622700 –KX622727, Cozzi et al., 2017), donkeys from Turkey and Cyprus (Cinar-Kul et al., 2016), ancestral Nubian wild donkeys (HM622634, HM622635, HM622636, Kimura et al., 2011), Somali wild donkeys (AY569545, AY569546, AY569547, Beja-Pereira et al., 2004) and a donkey reference sequence (X97337, Xu, Gullberg & Arnason, 1996), for assessing their genealogical relationship by constructing median-joining (MJ) network with NETWORK 4.6.1.2 (Bandelt, Forster & Rohl, 1999). The matrix comprising 71 mtDNA D-loop sequence was aligned using MUSCLE (Edgar, 2004) in MEGA4 (Tamura et al., 2007), and was truncated to match the length of the shortest available sequence (307 base pairs-bp). The parameter ε was kept at 0, and resulting network was manually assembled for visualization.

## Results

### Banat donkey population in Serbia

The largest number of individuals that differ morphologically from the Balkan donkey and, according to local breeders, represent the Banat donkey, is currently present at KO: 12 adult individuals (ten females, two males) and seven foals <3 years old, while only three adult females and one foal were recorded at SP. The Banat donkey is maintained together with the Balkan donkey in both KO and SP, and the mating between breeds is allowed at both localities. Local breeder from ZA, however, aimed at preventing interbreed mating in their freely roaming herd by separating females during the mating season, and thus, we used individuals from this locality in our study. At present, seven adult females, one adult male and five foals are found at ZA. This herd was initially established with 10 individuals some 25 years ago, and, over time, old animals have successively been replaced with younger ones, some of which were acquired from nearby local farmers. Altogether, the number of extant adult Banat donkey individuals in ZA, KO and SP is less than 100, with apparent domination of females (20 females vs. three males).

### Morphological characterization of the Banat donkey

All 18 morphological traits measured in individuals from four groups were homogeneous (CV ≤ 21%, Table 1). Mean values of all body measurements were larger in Banat donkeys than in hybrids and individuals from both sub-populations of the Balkan donkeys (Table 1).

A significant effect of donkey groups on variation of studied parameters was supported by both Hotelling-Lawley and Wilks approximate *F*-tests in MANOVA analysis. Hotelling’s T2 test revealed statistically significant difference between BanD and both sub-populations of the Balkan donkey (Table 2). Furthermore, Tukey’s test in one-way ANOVA analysis clearly separated BanD into a distinct homogeneous group by all studied morphological parameters except ear length (Table 3), and revealed that statistically significant differences between two sub-populations of the Balkan donkey were obtained for four parameters: back height, chest depth, hip height and wither height, which are highly correlated (see below). Contribution of expected variances of groups’ main effect to the total variance (i.e., the intraclass correlation coefficient reflecting the ratio between expected group variance and sum of expected group variances and errors) was highest for body weight and hip height (>60%), and lowest for chest width and ear length (<20%) (Fig. S2). Thus, the difference between groups was best demonstrated by two parameters, body weight and hip height.

In the PCA analysis, the first three PCs, which explain 75% of the total variance, were selected for further rotation (Table S1). The first rotated PC explains almost half of the total variance (34.4%), and nine out of 18 studied morphological parameters have their highest loadings with this PC. Since parameters body weight and hip height, which have the highest contribution of factor group to the total expected variance (Fig. S2), belong to the same PCA-group, they were considered to be strongly correlated. It is worth mentioning that this analysis revealed that morphological traits that describe body length and weight, and the size of the head and extremities are highly correlated (first PCA-group), as well as those related to the height of animals (PCA-group 2) and the size of the chest and croup (PCA-group 3).

**Figure 2:**
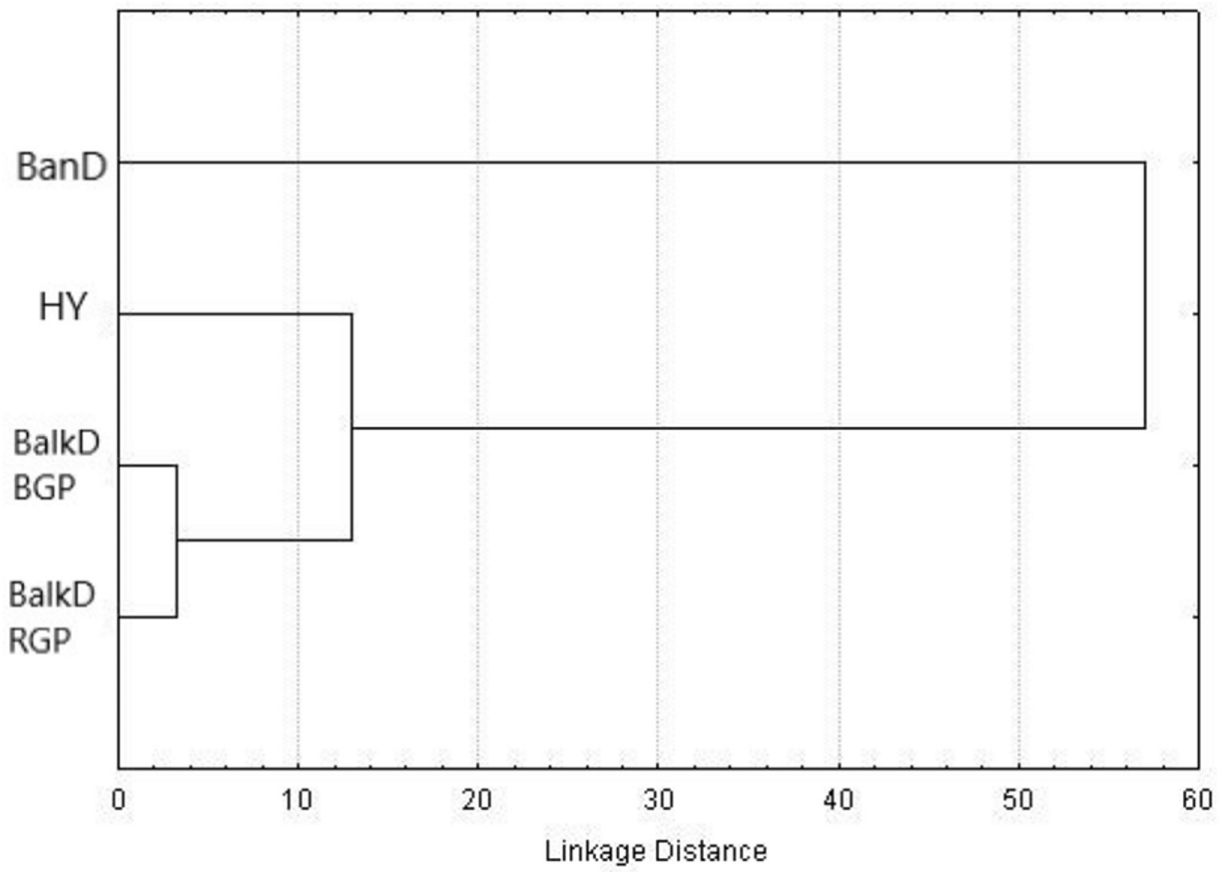
UPGMA dendrogram based on squared Mahalanobis distances between groups calculated using morphometric data. BanD, Banat donkey; HY, hybrid individuals; BalkD-BGP and BalkD-RGP, two sub-populations of the Balkan donkeys delineated based on their nuclear genetic profiles.

**Figure 3:**
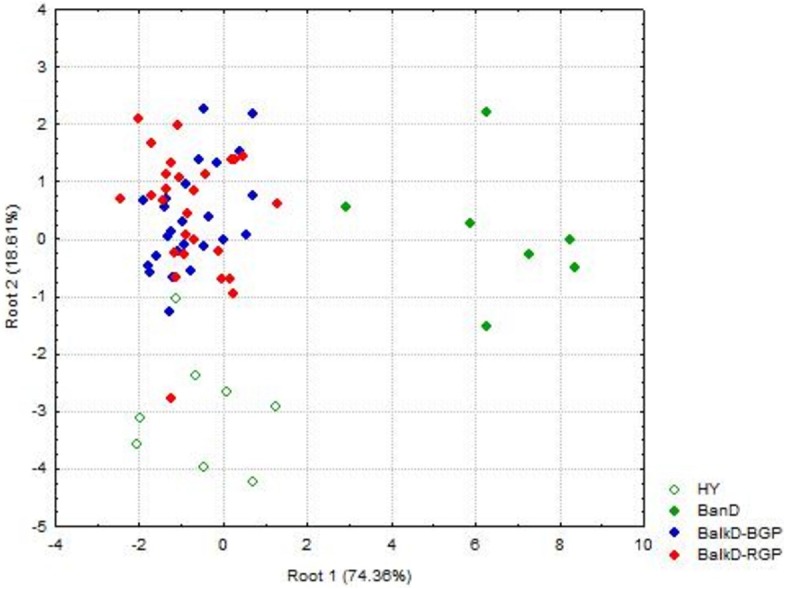
Canonical Discriminant Analysis of morphometric data. The scores of individuals from a particular group, demonstrating their relationships, are given in space defined by the first two canonical variables (roots). BanD, Banat donkey; HY, hybrid individuals; BalkD-BGP and BalkD-RGP, two sub-populations of the Balkan donkeys delineated based on their nuclear genetic profiles.

Morphological distinctiveness of Banat donkeys was evident also in the UPGMA dendrogram (Fig. 2) and in the Canonical Discriminant Analysis in which first two canonical variables jointly explain 93.0% of the total variance (Fig. 3). BanD was separated from other groups along the first canonical variable which explains most of the total variance (74.4%), while HY was separated from BalkD-BGP/BalkD-RGP along the second canonical variable which explains 18.6% of the total variance. The outcomes of this analysis are in accordance with the outcomes of the Hotelling’s T2 test which did not support significance of overall difference between two sub-populations of the Balkan donkey by morphological parameters.

**Table 1:** Sample size, average age of individuals in each group and measures of central tendencies, and variability of 18 morphological traits measured in a single Banat donkey male and females from four studied groups.

**#**	**Trait**	**BanDmale**	**Statisticalparameter**	**BanDfemales**	**HYfemales**	**BalkD-BGPfemales**	**BalkD-RGPfemales**
	**N**	1		7	8	26	27
	**Age**	8		5.25	5.75	5.27	5.03
			Mean (SD)	115.6 (6.1)	100.8 (3.7)	98.3 (6.2)	104.1 (5.3)
1	Back height	118	Median	117.0	102.5	97.5	104.0
			Quartile range	109.0–121.0	98.5–103.5	94.0–102.5	101.0–107.0
			CV (%)	5	4	6	5
			Mean (SD)	131.1 (6.9)	119.8 (6.0)	112.0 (6.7)	114.0 (6.3)
2	Body length	145	Median	132.0	122.0	113.0	115.0
			Quartile range	114.5–125.0	114.5–125.0	109.0–116.5	108.0–119.0
			CV (%)	5	5	6	6
			Mean (SD)	208.7 (28.0)	138.4 (12.0)	125.0 (22.8)	132.1 (18.2)
3	Body weight	219	Median	203.0	140	125.5	132.0
			Quartile range	190.0–245.0	133.0–146.5	115.0–142.0	118.0–149.0
			CV (%)	13	9	18	14
			Mean (SD)	25.1 (1.2)	20.6 (2.3)	20.6 (1.6)	21.1 (1.3)
4	Carpal circumference	29	Median	25.5	21.0	21.0	21.0
			Quartile range	24.0–26.0	18.5–21.7	19.5–22.0	20.0–22.0
			CV (%)	5	11	8	6
			Mean (SD)	37.3 (3.2)	29.8 (2.8)	31.4 (3.0)	32.7 (2.2)
5	Carpal height	41	Median	39.0	30.7	30.5	33.0
			Quartile range	36.0–39.0	28.0–32.0	30.0–30.5	31.0–34.0
			CV (%)	9	9	9	7
			Mean (SD)	137.4 (7.5)	117.2 (3.4)	114.7 (9.0)	117.1 (5.6)
6	Chest circumference	134	Median	138.5	117.5	114.5	117.0
			Quartile range	131.0–144.0	114.7–120.7	110.0–122.0	114.5–121.0
			CV (%)	5	3	8	5
			Mean (SD)	52.7 (3.1)	46.6 (2.5)	44.7 (3.6)	49.2 (3.9)
7	Chest depth	60	Median	52.0	46.7	44.7	50.0
			Quartile range	50.5–57.0	45.0–47.2	43.0–47.0	46.0–53.0
			CV (%)	6	5	8	8
			Mean (SD)	26.7 (3.1)	22.4 (2.9)	24.0 (2.6)	24.9 (2.0)
8	Chest width	28	Median	27.0	22.7	24.0	25.0
			Quartile range	24.0–30.0	19.5–24.0	22.5–26.0	24.0–26.0
			CV (%)	12	13	11	8
			Mean (SD)	38.9 (3.0)	27.5 (4.6)	28.0 (5.8)	31.0 (3.9)
9	Croup length	40	Median	38.0	26.5	26.0	32.0
			Quartile range	36.0–42.0	23.5–30.2	22.0–33.0	30.0–34.0
			CV (%)	8	17	21	12
			Mean (SD)	38.6 (3.7)	30.0 (1.8)	34.6 (3.8)	35.7 (3.1)
10	Croup width	43	Median	37.0	30.7	35.5	36.0
			Quartile range	35.0–43.0	28.5–31.2	33.0–37.0	34.0–38.0
			CV (%)	10	6	11	9
			Mean (SD)	27.0 (1.8)	24.7 (1.7)	25.1 (2.2)	26.3 (1.8)
11	Ear length	30	Median	27.0	25.0	25.5	26.0
			Quartile range	26.0–28.0	23.5–25.5	24.0–26.0	25.0–28.0
			CV (%)	7	7	9	7
			Mean (SD)	54.9 (2.0)	47.7 (2.7)	48.5 (3.1)	48.4 (2.6)
12	Head length	56	Median	55.0	47.0	48.7	48.0
			Quartile range	53.0–57.0	45.5–50.2	47.0–51.0	46.0–51.0
			CV (%)	4	6	6	5
			Mean (SD)	26.4 (3.1)	22.0 (2.3)	21.0 (1.5)	21.2 (1.2)
13	Head width	28	Median	28.0	23.0	21.0	21.0
			Quartile range	23.0–30.0	20.5–23.7	20.5–22.0	20.0–22.0
			CV (%)	12	10	7	6
			Mean (SD)	116.3 (6.2)	103.8 (4.0)	103.5 (6.2)	108.8 (5.8)
14	Hip height	131	Median	115.0	103.5	104.2	110.0
			Quartile range	112.0–121.0	101.2–107.7	99.0–108.0	105.0–112.0
			CV (%)	5	4	6	5
			Mean (SD)	34.0 (2.6)	28.4 (1.3)	27.2 (1.6)	26.0 (1.3)
15	Tarsal circumference	36	Median	34.0	28.5	27.0	28.0
			Quartile range	33.0–36.0	27.5–28.7	26.0–28.5	27.0–29.0
			CV (%)	8	4	6	4
			Mean (SD)	46.9 (2.1)	39.2 (3.2)	40.3 (3.0)	40.6 (2.7)
16	Tarsal height	46	Median	46.0	39.0	40.5	40.0
			Quartile range	45.0–49.0	36.5–41.5	38.5–42.5	38.0–43.5
			CV (%)	4	8	7	7
			Mean (SD)	29.7 (2.4)	24.7 (1.1)	24.1 (2.1)	24.1 (1.6)
17	Tibia circumference	34	Median	29.0	24.5	24.0	24.0
			Quartile range	27.0–33.0	24.0–25.5	23.0–26.0	23.0–25.0
			CV (%)	8	4	9	7
			Mean (SD)	117.9 (6.5)	103.2 (3.6)	100.1 (6.2)	105.9 (5.0)
18	Wither height	126	Median	121.0	104.5	99.0	109.0
			Quartile range	110.0–122.0	100.7–105.5	96.5–105.0	102.5–109.0
			CV (%)	5	4	6	5

**Notes.**

BanD Banat donkey HY hybrid individuals BalkD-BGP and BalkD-RGP two sub-populations of the Balkan donkeys delineated based on their nuclear genetic profiles

Values of all morphological traits are given in cm, except for Body weight given in kg.

N sample size Age average age given in years SD standard deviation CV coefficient of variation in %

**Table 2:** Hotelling’s T2 test demonstrating the significance of differences between studied groups based on 18 measured morphological traits.

**Group**	**BanD**	**HY**	**Balk-BGP**	**Balk-RGP**
BanD	–	a	13.368^**^	10.761^**^
HY		–	3.939^**^	3.056^*^
Balk-BGP			–	1.105
Balk-RGP				–

**Notes.**

BanD Banat donkey HY hybrid individuals BalkD-BGP and BalkD-RGP two sub-populations of the Balkan donkeys delineated based on their nuclear genetic profiles

a, test between BanD and HY was not performed because the sample size of each of these groups was lower than the number of measured parameters +1.

* *P* < 0.05.

** *P* < 0.01.

**Table 3:** One-way ANOVA and Tukey’s test used for assessing statistical significance of differences between studied groups based on ANOVA.

	**Variables**	**ANOVA**			**Tukey’s test**			
		**MS_groups_**	**MS_error_**	***F*-test**	**BanD**	**HY**	**Balk-BGP**	**Balk-RGP**
1	Back height (cm)	583.61	33.41	17.47^**^	115.57 a	100.81 bc	98.29 c	104.15 b
2	Body length (cm)	738.59	44.87	16.46^**^	131.07 a	119.75 b	112 c	114.04 bc
3	Body weight (kg)	13440.29	454.16	29.59^**^	208.71 a	138.38 b	125.04 b	132.07 b
4	Carpal circumference (cm)	38.77	2.52	15.37^**^	25.07 a	20.63 b	20.62 b	21.11 b
5	Carpal height (cm)	83.21	7.80	10.67^**^	37.29 a	29.81 b	31.38 b	32.7 b
6	Chest circumference (cm)	976.68	53.41	18.29^**^	137.36 a	117.25 b	114.67 b	117.13 b
7	Chest depth (cm)	150.46	13.35	11.27^**^	52.71 a	46.63 bc	44.87 c	49.17 ab
8	Chest width (cm)	26.71	6.47	4.13^**^	26.71 a	22.44 b	24 ab	24.94 ab
9	Croup length (cm)	242.47	23.40	10.36^**^	38.86 a	27.5 b	28 b	31.04 b
10	Croup width (cm)	103.70	11.83	8.76^**^	38.64 a	30 c	34.58 b	35.72 ab
11	Ear length (cm)	13.28	4.17	3.18^*^	27 a	24.75 a	25.1 a	26.35 a
12	Head length (cm)	89.36	8.16	10.95^**^	54.86 a	47.69 b	48.5 b	48.46 b
13	Head width (cm)	59.06	3.24	18.21^**^	26.43 a	22 b	20.98 b	21.2 b
14	Hip height (cm)	362.99	36.30	10^**^	116.29 a	103.81 bc	103.54 c	108.85 b
15	Tarsal circumference (cm)	88.93	2.62	33.9^**^	34 a	28.38 b	27.17 b	27.81 b
16	Tarsal height (cm)	93.50	8.29	11.28^**^	46.86 a	39.25 b	40.33 b	40.61 b
17	Tibia circumference (cm)	64.25	3.66	17.58^**^	29.71 a	24.69 b	24.12 b	24.15 b
18	Wither height (cm)	608.72	32.29	18.85^**^	117.86 a	103.19 bc	100.06 c	105.87 b

**Notes.**

BanD Banat donkey HY hybrid individuals BalkD-BGP and BalkD-RGP two sub-populations of the Balkan donkeys delineated based on their nuclear genetic profiles MS_groups_ mean squares of factor groups MS_error_ mean squares of Error

a, b and c denote homogeneous groups, where differences between group average values of a particular trait with the same letter are not significant.

* *P* < 0.05.

** *P* < 0.01.

**Figure 4:**
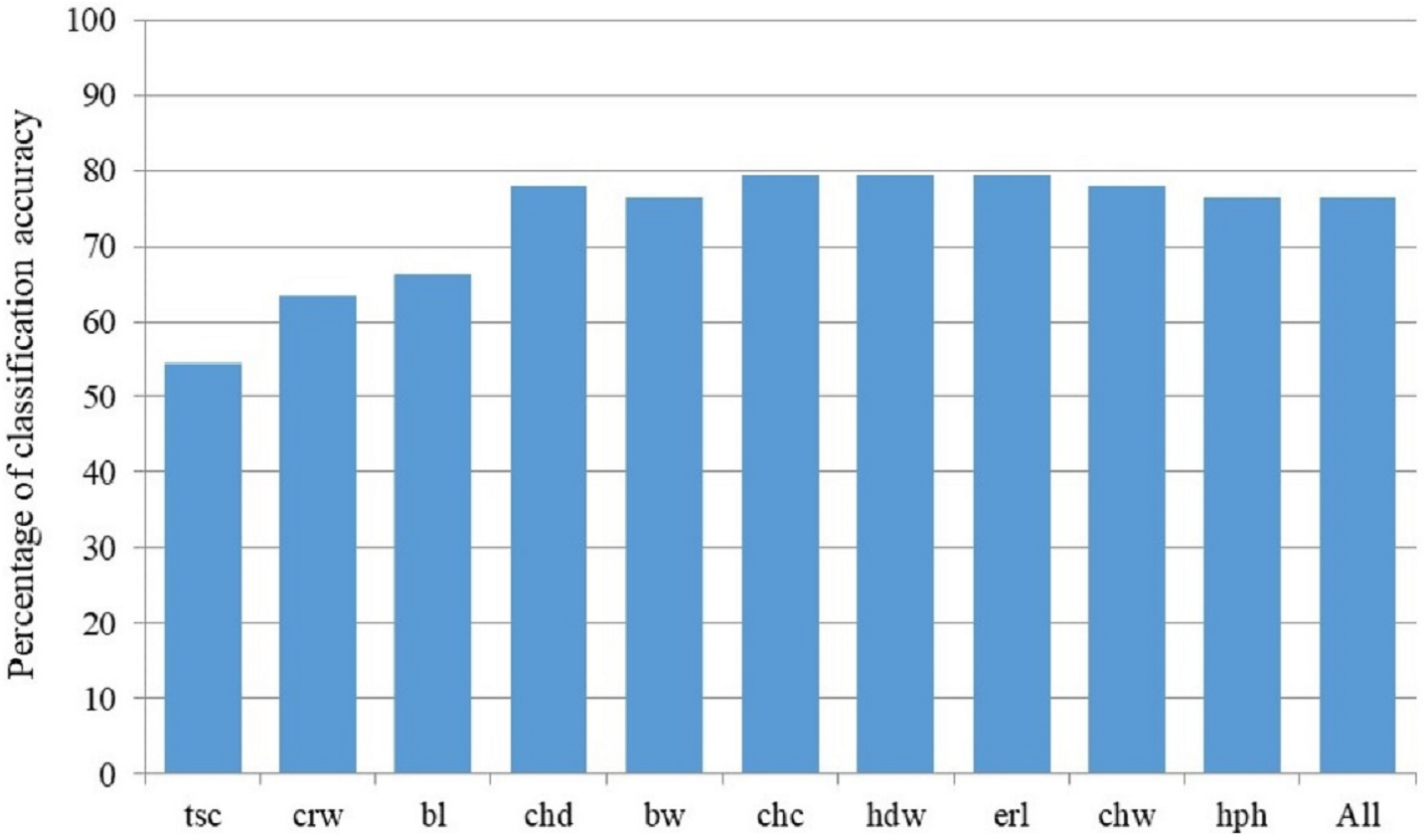
Percentage of classification accuracy of successive models derived by Stepwise Forward Discriminant Analysis. Label of the last included parameter in corresponding discriminant model on *X*-axis: bl, Body length (cm); bw, Body weight (kg); chc, Chest circumference (cm); chd, Chest depth (cm); chw, Chest width (cm); crw, Croup width (cm); erl, Ear length (cm); hdw, Head width (cm); hph, Hip height (cm); tsc, Tarsal circumference (cm); All stands for the model with all studied morphological parameters included.

Discriminant model based on all 18 measured parameters achieved 76.5% of classification accuracy, and Stepwise Forward Discriminant Analysis procedure revealed that this accuracy could be also achieved by the model that includes only four parameters: hip height, croup width, body length and chest depth (Fig. 4). Taking into account the loadings of these parameters with first three rotated PCs in the PCA analysis (Table S1), it is evident that they belong to different PCA-groups, i.e., hip height and body length to the first, croup width to the second, and chest depth to the third PCA-group.

### Genetic characterization of the Banat donkey

We take advantage of the dataset of Stanisic et al. (2017) (genetic profiles of BanD, HY, BalkD-BGP and BalkD-RGP assessed with 11 microsatellites from the Equine Genotype Panel 1.1 recommended by the International Society for Animal Genetics (ISAG) for equine parentage and identification testing) to characterize genetically Banat donkey and to compare four donkey groups.

The parameters of genetic diversity of Banat donkey, represented by seven females, were as follows: *A* = 58, *Ae* = 3.749 ± 0.255, *H*_*O*_ = 0.799 ± 0.075, and *H*_*E*_ = 0.777 ± 0.024 (Table 4). Two private alleles were found in this group characterized also by the significant excess of heterozygotes (*F*_*IS*_ =  − 0.111 ± 0.095).

**Table 4:** Parameters of genetic diversity in four studied groups based on variability of 11 nuclear microsatellites.

	**BanD**	**HY**	**BalkD-BGP**	**BalkD-RGP**
*N*	7	8	26	27
*A*	58	58	82	69
*PA*	2	3	14	2
*Ae* (SE)	3.749 (0.255)	3.587 (0.363)	4.654 (0.545)	3.528 (0.284)
*H*_*O*_ (SE)	0.799 (0.075)	0.650 (0.088)	0.772 (0.063)	0.853 (0.052)
*H*_*E*_ (SE)	0.777 (0.024)	0.719 (0.058)	0.775 (0.026)	0.712 (0.026)
*F*_*IS*_ (SE)	−0.111^*^ (0.095)	0.081 (0.127)	−0.013 (0.067)	−0.221^*^ (0.056)

**Notes.**

BanD Banat donkey HY hybrid individuals BalkD-BGP and BalkD-RGP two sub-populations of the Balkan donkeys delineated based on their nuclear genetic profiles N sample size A number of alleles PA number of private alleles *Ae* effective number of alleles *H*_*O*_ observed heterozygosity *H*_*E*_ expected heterozygosity *F*_*IS*_ inbreeding coefficient SE standard error

* *P* < 0.05.

** *P* < 0.01.

**Figure 5:**
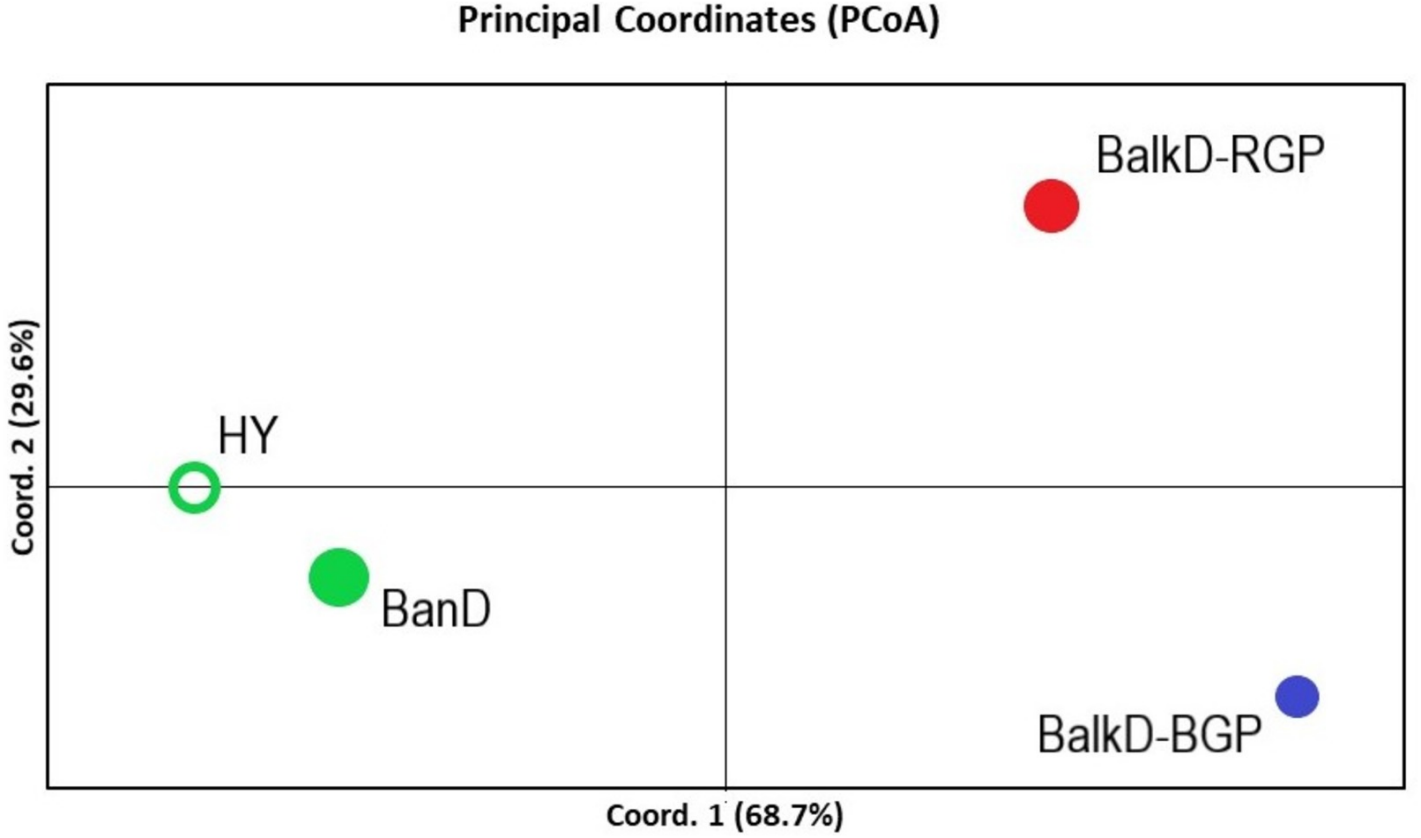
PCoA analysis based on nuclear microsatellite data. The scores of four studied groups are given in the space defined by the first two principal coordinates. BanD, Banat donkey; HY, hybrid individuals; BalkD-BGP and BalkD-RGP, two sub-populations of the Balkan donkeys delineated based on their nuclear genetic profiles.

**Figure 6:**
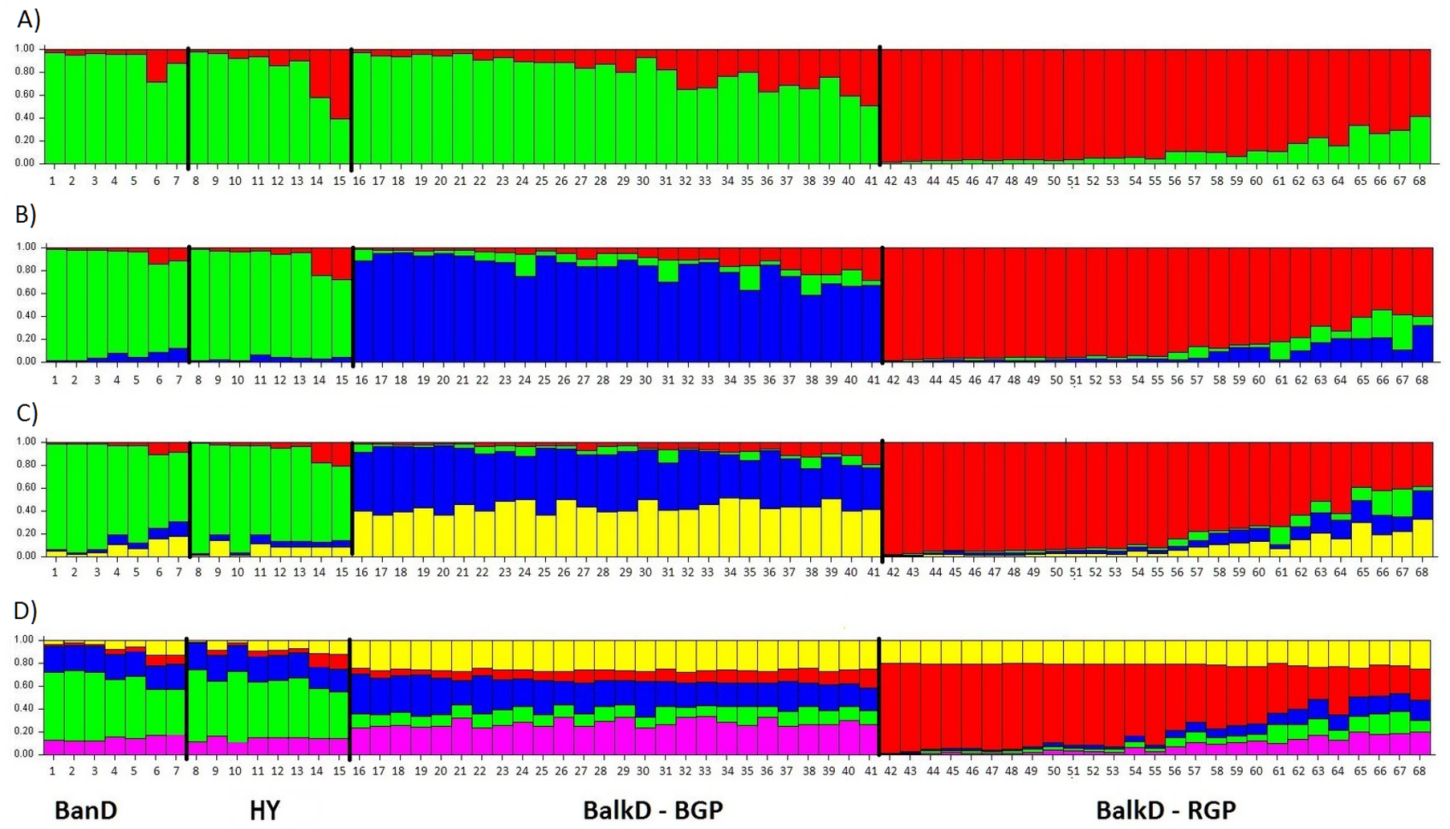
Bayesian clustering as determined by STRUCTURE analysis of nuclear microsatellite data at *K* = 2 − 5. (A) Structure with *K* = 2; (B) Structure with *K* = 3; (C) Structure with *K* = 4; (D) Structure with *K* = 5. BanD, Banat donkey; HY, hybrid individuals; BalkD-BGP and BalkD-RGP, two sub-populations of the Balkan donkeys delineated based on their nuclear genetic profiles.

All pairwise-group *F*_*ST*_ values were statistically significant at 95% level (Table 5). *F*_*ST*_ value between Banat donkeys and potential hybrids was in the range of that observed between the two sub-populations of the Balkan donkey (0.044 vs. 0.037), while those between Banat donkeys and each of the two sub-populations of the Balkan donkey were almost doubled (Table 5). Concordantly, the highest number of migrants per generation, reflecting past gene flow, was observed between Banat donkeys and potential hybrids (*Nm* = 5.38) and between the two sub-populations of the Balkan donkey (*Nm* = 6.44). In MDS graph (Fig. S3), Banat donkeys and potential hybrids were separated from the two sub-populations of the Balkan donkey along the first dimension, and the same grouping was observed in the PCoA graph defined with axis 1 (explaining 68.70% of variability) and axis 2 (explaining 29.60% of variability) (Fig. 5). A small but statistically significant portion of the total genetic variability was allocated to the among-population component (6.63%, *P* = 0.001, Table S2). The outcomes of the Bayesian clustering analysis (Fig. 6) were concordant with those reported previously by Stanisic et al. (2017), with optimal number of three genetic groups (Fig. S4). Banat donkeys and potential hybrids were assigned to the unique green gene pool, and they kept their genetic integrity in analyses at *K* = 3–5. The same pattern was observed for Balkan donkey individuals assigned to the red gene pool (RGP), while those assigned to the blue gene pool (BGP) displayed admixed nuclear genetic profiles at *K* = 4–5.

**Table 5:** Pairwise populations *F*_*ST*_ values (below diagonal) and number of migrants per generation (above diagonal) based on variability of 11 nuclear microsatellites.

	**BanD**	**HY**	**BalkD-BGP**	**BalkD-RGP**
BanD	0	5.38	4.13	3.72
HY	0.044^***^	0	3.14	3.33
BalkD-BGP	0.057^***^	0.074^***^	0	6.44
BalkD-RGP	0.063^***^	0.070^***^	0.037^***^	0

**Notes.**

BanD Banat donkey HY hybrid individuals BalkD-BGP and BalkD-RGP two sub-populations of the Balkan donkeys delineated based on their nuclear genetic profiles

* *P* < 0.05.

** *P* < 0.01.

*** *P* < 0.001.

### Genealogical relationship of the mtDNA haplotypes of the Banat and other studied donkeys

**Figure 7:**
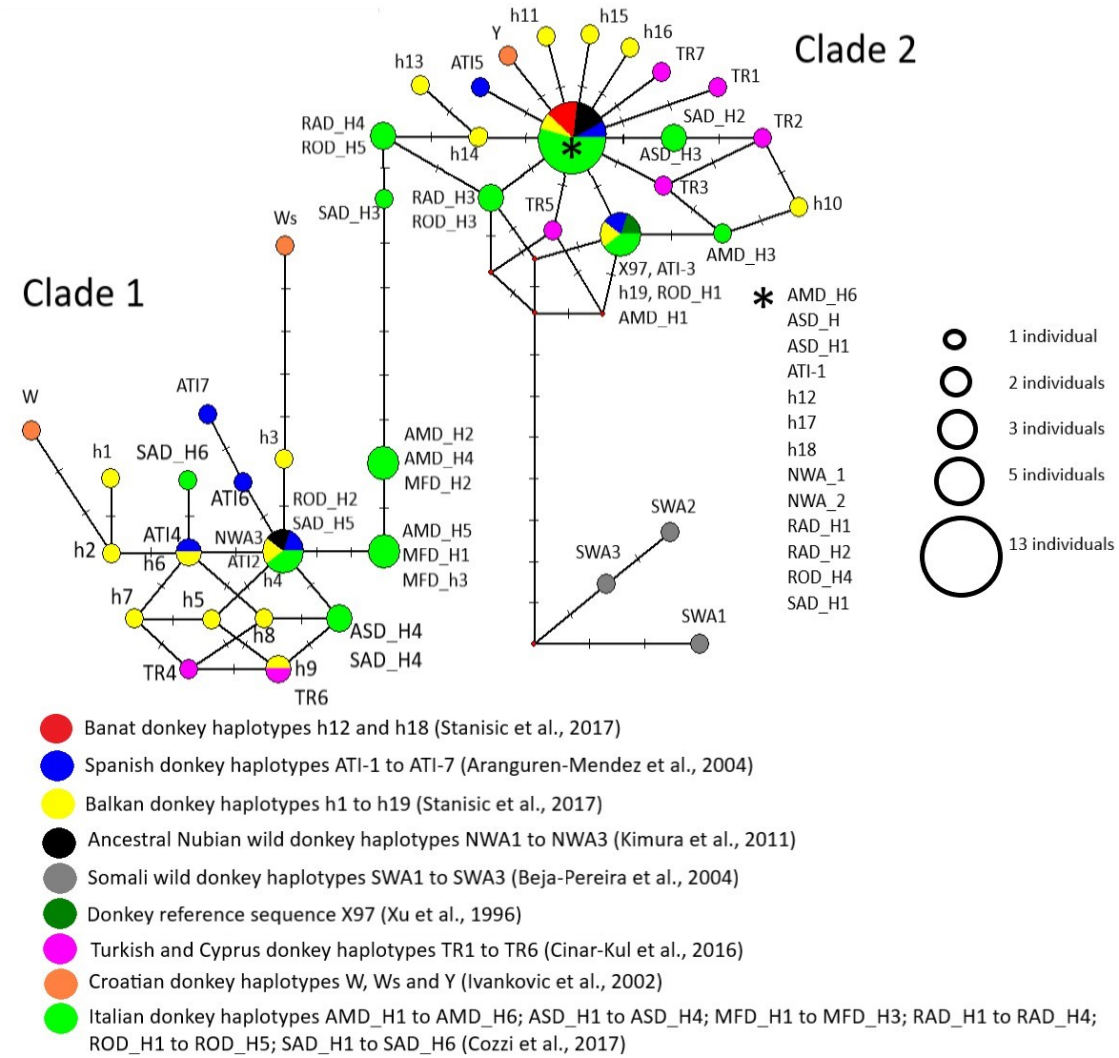
Median-Joining network demonstrating genealogical relations of 71 mitochondrial haplotypes assessed based on the variability of the mtDNA D-loop.

Majority of studied mtDNA haplotypes were grouped into two lineages corresponding to Clades 1 and 2 defined previously by Beja-Pereira et al. (2004) and Kimura et al. (2011), while those found in Somali wild donkeys were allocated to the third lineage which was genealogically linked to Clade 2 (Fig. 7). Since we used somewhat shorter sequences than those used for the delineation of distinct mtDNA haplotypes in corresponding studies, haplotypes distinguished from each other in those studies were sometimes presented jointly within the same node in our MJ network. For instance, haplotypes RAD-H4 and ROD-H5 found in two Italian donkey breeds occupy the same node because the mutation that distinguishes them was not present in our aligned matrix that was truncated. The node positioned in the centre of Clade 2 comprised two ancient Nubian wild donkey haplotypes, haplotypes h12, h17 and h18 found in Banat and Balkan donkeys, ATI-1 typical for Catalana, Mallorqina and Zamorano-Leonessa breeds from Spain and for two donkey populations from Africa (one from Morocco and the second one from Zimbabwe), and haplotypes found in individuals belonging to five donkey breeds from Italy (AMD-H6 present in Amiata donkeys, ASD-H1 and ASD-H2 found in Asinara donkeys, RAD-H1 and RAD-H2 found in Ragusano donkeys, ROD-H4 from Romagnolo donkey, and SAD-H1 from Sardo donkeys).

## Discussion

### Banat donkey population in Serbia

The donkey breed that is distributed today throughout the Balkan Peninsula and represented by a relatively small number of individuals is the heterogeneous Balkan donkey (Stanisic et al., 2017). However, additional donkey breeds may also be present in this large region, such as those found in Croatia (Istrian and North-Adriatic donkeys, Ivankovic et al., 2002) and in the north-eastern part of Serbia, the Banat region. The latter, the Banat donkey, is at present an uncharacterized and highly neglected donkey breed in Serbia. For instance, the common practice of local breeders at three localities in Serbia where large donkey herds are currently found, KO, SP and ZA, was to maintain a couple of individuals that differ from the prevalent Balkan donkey in order to demonstrate the morphological variability of donkeys to the people visiting their farms. Furthermore, over the past 25 years mating between the breeds was mainly allowed in KO and SP, and in a somewhat more limited fashion in ZA. Since in ZA we found potential hybrid individuals that are morphologically indistinguishable from the Balkan donkey and are characterized by a distinct nuclear gene pool (Stanisic et al., 2017; this study), it is evident that well-planned actions aimed at the conservation of this valuable genetic resource are urgently needed.

We found that, out of 281 sexually mature individuals registered in 2015 at KO, SP and ZA, 23 animals may be delineated as the Banat donkey based on their morphological features. Due to such a low number of Banat donkeys in Serbia, this breed may be characterized as critically endangered (FAO, 2015). However, according to local breeders, one or two Banat donkeys per household are still present in the rural parts of Vojvodina, the northern part of Serbia comprising Banat, Bačka and Srem districts. Therefore, future efforts of breed conservation should aim not only to preserve the original traits of the Banat donkey, through well-planned mating strategies in maintained herds (Kugler, Grunenfelder & Broxham, 2008), but also to acquire such individuals from local farmers. These individuals should be used for mating in order to prevent loss of genetic diversity and inbreeding through mating among relatives in small-sized populations (e.g., Gutiérrez et al., 2005; Quaresma et al., 2014). Furthermore, the search for Banat donkeys should encompass a larger area, preferably the entire Balkan region, because of the common transfers of donkeys throughout it. Given the general lack of controlled mating of donkeys in the past in the Balkans (Kugler, Grunenfelder & Broxham, 2008; Stanisic et al., 2015; Stanisic et al., 2017), all individuals should be characterised both morphologically and genetically in order to be assigned unambiguously to the Banat donkey breed.

### Morphological characterization of the Banat donkey

Comparative analysis of the 18 morphological traits of Banat and Balkan donkeys revealed that Banat donkeys are >70 kg heavier, >10 cm taller and >15 cm longer than the potential hybrids and individuals from both sub-populations of the Balkan donkey. Their chest circumference is >20 cm wider, and their hips and withers are 8–15 cm higher than those measured in other studied groups. Body measurements of a single studied 8-years old Banat donkey male (Table 1) generally correspond to those recorded for younger females (average age 5.25 years), with a somewhat larger difference observed in body length, carpal circumference, chest depth, hip height and tibia circumference, which are the most common morphological traits used for donkey breed characterization (e.g., Folch & Jordana, 1997; Sargentini et al., 2009; Sargentini et al., 2018; Kefena et al., 2011; Kosťuková et al., 2015; Labbaci et al., 2018; Ayad et al., 2019). This would indicate a possible sexual dimorphism in the Banat donkey which is indeed present in certain donkey breeds (e.g., Catalonian donkey from Spain, Folch & Jordana, 1997). For instance, Folch & Jordana (1997) found that the greatest difference between Catalonian donkey sexes is at the cephalic level, and also in height from the posterior third of the body and extremities perimeter, indicating that females are more elevated and more slender than males. However, lack of sexual dimorphism or explicit anatomical dissimilarities between the sexes is also common in donkeys (e.g., six Ethiopian donkey populations, Kefena et al., 2011), and thus, inferences on the possible Banat donkey’s sexual dimorphism require analyses of additional males.

A profound morphological difference between the Banat and Balkan donkey was demonstrated in all the performed statistical analyses, suggesting that the Banat donkey may be acknowledged as a distinct breed based on its morphological features (Alderson, 2003). The most important parameters for the separation of the Banat donkey from the Balkan donkey and potential hybrids are body weight and hip height, which however are highly correlated. We also found that the classification accuracy in the Stepwise Forward Discriminant Analysis obtained with the model that included all studied morphological traits may also be achieved by applying the model that includes only four traits: hip height, croup width, body length and chest depth. These results highlight the redundancy of the majority of morphological traits used in our study, which are, more or less, similar to those commonly used for morphological characterization of donkey breeds (e.g., Folch & Jordana, 1997; Sargentini et al., 2009; Sargentini et al., 2018; Kefena et al., 2011; Kosťuková et al., 2015; Labbaci et al., 2018; Ayad et al., 2019). Nonetheless, an important finding is that hip height/chest depth, croup width and body length are weakly correlated because these parameters (or parameters with which they are highly correlated) belong to different PCA-groups (Table S1). This would suggest that all these parameters should be used for assessing morphological distinctiveness of Banat and Balkan donkey breeds. It is worth mentioning that we found that two sub-populations of the Balkan donkey, which are distinct at the genetic level (Stanisic et al., 2017; this study), may be distinguished at the morphological level as well, based on one of the four highly correlated parameters, namely back height, chest depth, hip height and wither height.

The fact that extant Banat donkeys are taller, longer, somewhat wider and much heavier than Balkan donkey is in line with speculations of local breeders from Serbia that they are descendants of the heard of more robust, larger and stronger Spanish donkeys transferred from Spain to the Banat region ∼300 years ago for a specific purpose, namely to help farmers work the local vineyards. Their body constitution was apparently maintained by more or less controlled mating over time despite the general lack of such management practice in Serbia (Stanisic et al., 2015) and worldwide (Kugler, Grunenfelder & Broxham, 2008). However, the discovery of a potential hybrid population in ZA, where Banat donkeys are kept together with Balkan donkeys (Stanisic et al., 2017), highlights the need for urgent preservation of the Banat donkey and maintenance of its original traits through well-planned mating strategies (Kugler, Grunenfelder & Broxham, 2008). It is noteworthy that Banat donkey females produce greater quantities of quality milk than Balkan donkey females (∼30 l, data acquired from local breeders), implying that maintenance of this breed by local breeders may be economically beneficial.

Although further genetic studies are required to decipher whether the black-coated Catalonian donkey from the north-eastern Spain (Folch & Jordana, 1997) was indeed introduced to the Banat region, this breed has been commonly used in the past for the improvement of numerous European and American donkey breeds (Jordana & Folch, 1996). The mating between large-sized Catalonian donkey (Folch & Jordana, 1997) and mid-sized and lighter coloured Balkan donkeys (Stanisic et al., 2015) may account for the successive decrease in size and change in the coat colour in extant Banat donkeys. A unique morphological feature of the Banat donkey, black stripes on legs resembling those typical for Somali wild donkeys (Groves, 1986; Moehlman, 2002) present today in Somalia, Ethiopia and Eritrea (Moehlman, 2002), may represent a relict and primitive marking (Johnson & Johnson, 2008) indicative of their origin. According to Sargentini et al. (2018), black stripes on legs and some other body markings characterize also the Italian Amiata donkey breed and their ancestors.

### Genetic characterization of the Banat donkey

Although in this study we used genetic profiles at the nuclear DNA level of only seven adult Banat donkey females, all the parameters of genetic diversity calculated for this group were similar or even higher compared to those observed in the two larger sub-populations of Balkan donkey females (Table 4), and also in populations of other donkey breeds distributed worldwide (Jordana, Folch & Sanchezm, 1999; Jordana et al., 2016; Aranguren-Mendez, Jordana & Gomez, 2001; Ivankovic et al., 2002; Blasi et al., 2005; Ciampolini et al., 2007; Zhu et al., 2013; Matassino et al., 2014; Rosenbom et al., 2015). Furthermore, two private alleles were observed in Banat donkeys characterized also by the significant excess of heterozygotes. Therefore, although small, the female Banat donkey population currently kept in ZA is not severely affected by the loss of genetic diversity or by inbreeding. The hybrid group of eight individuals found in ZA is characterized by slightly lower levels of genetic diversity, three private alleles, and statistically insignificant excess of homozygotes. Since they are morphologically indistinguishable from Balkan donkeys but mainly harbour Clade 2 mtDNA haplotypes which are dominant in Banat donkeys, they most likely emerged as a result of mating between Banat donkey females with Balkan donkey males which are abundant in ZA. However, the scores of these individuals in the PCoA plot (Fig. 5) were not intermediate between the Banat and the Balkan donkey, and thus we cannot exclude the possibility of genetic input from sources other than the Balkan donkey in these donkeys. This is supported by the presence of three private alleles in these eight individuals. Given the fact that Banat donkey individuals currently present in ZA were acquired from local farmers which did not keep records on individuals which were used for mating with Banat donkey, the genetic source accounting for the positioning of the score of the hybrid group in PCoA graph remains unknown. Nonetheless, it is very likely that these individuals that display genetic affinity towards Banat donkey and morphological similarity with the Balkan donkey are hybrids with rather complex and unknown ancestry.

Genetic distinctiveness of the Banat donkey was supported by all the analyses of genetic differentiation that were performed in the course of our study. Thus, we support the previous findings of Stanisic et al. (2017) that the Banat donkey differs genetically from the Balkan donkey, and that it may be acknowledged as a distinct donkey breed in Serbia, the Balkans and worldwide (Alderson, 2003). A group of seven Banat donkey females represents a good core population which can be enlarged by introduction of unrelated individuals, both females and males, required for maintaining high levels of genetic diversity essential for successful preservation of the breed (e.g., Gutiérrez et al., 2005; Quaresma et al., 2014). Management practice in ZA should be improved in order to limit the mating between different breeds and enable preservation of original Banat donkey traits (Kugler, Grunenfelder & Broxham, 2008).

### Genealogical relationship among the mtDNA haplotypes of the Banat and other studied donkeys

Although our mtDNA data do not have the power to fully resolve the genealogical relationship among the mtDNA haplotypes used in our study, the finding that haplotypes h12 and h18 which are prevalent in the Banat donkey occupy the same node with haplotype ATI-1 typical for Catalana, Mallorqina and Zamorano-Leonessa breeds from Spain and for two donkey populations from Africa (one from Morocco and the other from Zimbabwe), would indeed indicate their close genealogical relationship. On the other hand, the presence of two ancient Nubian wild donkeys’ haplotypes in the same node in MJ network may imply a relict nature of mtDNA haplotypes found in extant Banat donkeys. Thus, the Banat donkey may indeed represent a breed that was established over time in the new environment, the Banat region in Serbia, from a herd that may have been introduced from Spain some 300 years ago. According to Aranguren-Mendez et al. (2004), mtDNA haplotypes ATI-1 and ATI-3, shared by three Spanish donkey breeds and two African donkey populations in more or less similar proportions, represent the most ancestral mtDNA types.

Recent molecular evidence questions the hypothesis of Beja-Pereira et al. (2004) and Kimura et al. (2011) that extinct Somali wild donkeys were the ancestors of the Clade 2 donkeys (e.g., Kefena et al., 2014; Stanisic et al., 2017; Xia et al., 2019) and, in addition, puts forward alternative centres of origin of donkeys belonging to this lineage (Rosenbom et al., 2015). Our data, however, would imply that the origin of Clade 2 donkeys may be much more complex than previously thought. For instance, we found also that a number of haplotypes found in individuals belonging to several donkey breeds from Italy (including the Amiata breed, Cozzi et al., 2017) are positioned within the same central node of Clade 2 as Spanish ATI-1 haplotype, indicating a close genealogical relationship of donkeys from Iberian Peninsula and Italian donkeys reported previously by Cozzi et al. (2017). Also, Spanish ATI-1 haplotype is found in Anatolian and donkeys from the Cyprus (Cinar-Kul et al., 2016). Therefore, our data support a close genealogical relationship of donkeys from all three south European Peninsulas, Turkey/Cyprus and Africa (i.e., the Mediterranean region), and furthermore, reveal that haplotypes found in several Italian donkeys occupy an intermediate position at the backbone of the MJ network between Clade 1 and Clade 2 haplotypes. These haplotypes of Italian donkeys were not available and were not included into the study of Stanisic et al. (2017) in which an intermediate positions between two Clades were occupied mainly by haplotypes found in donkeys from Albania, Bulgaria and Croatia.

Regarding the Banat donkey, which is characterized by the high proportion of Clade 2 mtDNA haplotypes, this breed may indeed represent a lineage evolving from Spanish matrilineal lineages, while a unique morphological feature shared between the Banat donkey, the Amiata donkey (Sargentini et al., 2009; Sargentini et al., 2018) and the Somali wild donkeys (Groves, 1986; Moehlman, 2002) may imply their common origin.

## Conclusions

We demonstrate that the neglected Banat donkey, nowadays traditionally maintained in the north-eastern part of Serbia, the Banat region, differs morphologically and genetically from the Balkan donkey, and may be acknowledged as a distinct donkey breed in Serbia, the Balkans and worldwide (Alderson, 2003). Following the FAO criteria for classifying the degree of endangerment of a breed, the Banat donkey, represented today by <100 living individuals (adults and foals), should be characterized as critically endangered (FAO, 2015). Therefore, region-wide actions concerning breed preservation, including well-planned mating strategies needed for the maintenance of the original traits of the breed (Kugler, Grunenfelder & Broxham, 2008), are urgently needed. In addition, we support the speculations of local breeders from Serbia regarding the Spanish origin of individuals giving rise to this breed in the new environment in the Balkans, because the Banat donkey shares mtDNA haplotypes with Spanish Catalana, Mallorqina and Zamorano-Leonessa breeds. Given the ancestral nature of these mtDNA haplotypes, the Banat donkey may represent a valuable genetic resource. A unique morphological feature shared between the Banat donkey, the Italian Amiata donkey and the Somali wild donkeys suggests that the origin of Clade 2 donkeys may be much more complex than previously thought.

##  Supplemental Information

10.7717/peerj.8598/supp-1 Table S1 Loadings of morphological parameters with the first three rotated principal components (RC) Underlined values given in bold represent the highest loading of a particular measured parameter on a given rotated principal component.

10.7717/peerj.8598/supp-2 Table S2 AMOVA analysis based on variability of 11 nuclear microsatellites in four studied groups d.f., degrees of freedom. *P < 0.05; **P < 0.01; ***P < 0.001.

10.7717/peerj.8598/supp-3 Figure S1 Schematic diagram showing major morphological variables measured on donkeys Abbreviations of measured morphological traits: BcH, Back height (cm); BL, Body length (cm); bw, Body weight (kg); CrC, Carpal circumference (cm); CrH, Carpal height (cm); ChC, Chest circumference (cm); ChD, Chest depth (cm); ChW, Chest width (cm); CrL, Croup length (cm); CrW, Croup width (cm); ErL, Ear length (cm); HdL, Head length (cm); HdW, Head width (cm); HpH, Hip height (cm); TsC, Tarsal circumference (cm); TsH, Tarsal height (cm); TbC, Tibia circumference (cm); WtH, Wither height (cm).

10.7717/peerj.8598/supp-4 Figure S2 Contribution of expected variances of group effect to the total variance of examined morphological traits Abbreviations of measured morphological traits: bkh, Back height (cm); bl, Body length (cm); bw, Body weight (kg); crc, Carpal circumference (cm); crh, Carpal height (cm); chc, Chest circumference (cm); chd, Chest depth (cm); chw, Chest width (cm); crl, Croup length (cm); crw, Croup width (cm); erl, Ear length (cm); hdl, Head length (cm); hdw, Head width (cm); hph, Hip height (cm); tsc, Tarsal circumference (cm); tsh, Tarsal height (cm); tbc, Tibia circumference (cm), wth, Wither height (cm). Groups are Banat donkey (BanD), potential hybrids (HY), and two sub-populations of the Balkan donkey, BalkD-BGP and BalkD-RGP.

10.7717/peerj.8598/supp-5 Figure S3 MDS graph with visual presentation of the matrix of pairwise group *F*_*ST*_ values. Stress value is zero. Groups are Banat donkey (BanD), potential hybrids (HY), and two sub-populations of the Balkan donkey, BalkD-BGP and BalkD-RGP

10.7717/peerj.8598/supp-6 Figure S4 Inferring optimal number of genetic clusters from STRUCTURE analysis based on distribution of Pr(K) at *K* ranging from 1 to 6

## Additional Information and Declarations

### Data Availability

The following information was supplied regarding data availability: Data is available at Figshare: Aleksic J, Stanisic LJ, Dimitrijevic V, Kovacevic B, Stevanovic J, Stanimirovic Z. 2019. Morphometric data and microsatellite profiles of 68 donkeys from 3 localities from Serbia (central Balkans) belonging to 2 donkey breeds, Banat donkey and Balkan donkey, and their hybrids. figshare. DOI: 10.6084/m9.figshare.10053299.v1.
