# Supplementary material for: Banat donkey, a neglected donkey breed from the central Balkans (Serbia)
Source: PeerJ. 2020 Mar 3;8:e8598. doi: 10.7717/peerj.8598 (PMC7059758; doi:10.7717/peerj.8598)
Supplement: Table S1 — Underlined values given in bold represent the highest loading of a particular measured parameter on a given rotated principal component. [file peerj-08-8598-s001.docx]

**Table S1** Loadings of morphological parameters with the first three rotated principal components (RC). Groups are Banat donkey (BanD), potential hybrids (HY), and two sub-populations of the Balkan donkey (BalkD-BGP and BalkD-RGP).

|  |  | **Rotated principal components** | | |
| --- | --- | --- | --- | --- |
|  | **Morphological trait** | **RC1** | **RC2** | **RC3** |
| 1 | Back height (cm) | 0.497 | **0.770** | 0.273 |
| 2 | Body length (cm) | **0.731** | 0.398 | 0.193 |
| 3 | Body weight (kg) | **0.783** | 0.450 | 0.204 |
| 4 | Carpal circumference (cm) | **0.701** | 0.335 | 0.439 |
| 5 | Carpal height (cm) | 0.436 | 0.437 | **0.520** |
| 6 | Chest circumference (cm) | **0.704** | 0.449 | 0.241 |
| 7 | Chest depth (cm) | 0.255 | **0.852** | 0.259 |
| 8 | Chest width (cm) | 0.211 | 0.039 | **0.798** |
| 9 | Croup length (cm) | 0.385 | 0.366 | **0.584** |
| 10 | Croup width (cm) | 0.151 | 0.360 | **0.741** |
| 11 | Ear length (cm) | 0.170 | **0.725** | 0.067 |
| 12 | Head length (cm) | **0.725** | 0.187 | 0.454 |
| 13 | Head width (cm) | **0.757** | 0.168 | 0.191 |
| 14 | Hip height (cm) | 0.386 | **0.794** | 0.348 |
| 15 | Tarsal circumference (cm) | **0.854** | 0.277 | 0.121 |
| 16 | Tarsal height (cm) | **0.654** | 0.325 | 0.276 |
| 17 | Tibia circumference (cm) | **0.797** | 0.204 | 0.189 |
| 18 | Wither height (cm) | 0.524 | **0.765** | 0.257 |
|  | **Explained variance** | 6.194 | 4.478 | 2.829 |
|  | **Proportion of explained in total variance** | 0.344 | 0.249 | 0.157 |

Underlined values given in bold represent the highest loading of a particular measured parameter on a given rotated principal component.
