# Supplementary material for: Banat donkey, a neglected donkey breed from the central Balkans (Serbia)
Source: PeerJ. 2020 Mar 3;8:e8598. doi: 10.7717/peerj.8598 (PMC7059758; doi:10.7717/peerj.8598)
Supplement: Table S2 — d.f., degrees of freedom. *P < 0.05; **P < 0.01; ***P < 0.001. [file peerj-08-8598-s002.docx]

**Table S2.** AMOVA analysis based on variability of 11 nuclear microsatellites in four studied groups (Banat donkey, BanD, potential hybrids, HY, and two sub-populations of the Balkan donkey, BalkD-BGP and BalkD-RGP).

| **Source of variation** | **d.f.** | **Sum of squares** | **Variance components** | **% of variation** | **Fixation indices** | ***P-*value** |
| --- | --- | --- | --- | --- | --- | --- |
| Among population | 3 | 22.750 | 0.178 | 6.63 | *F_ST_* = 0.066*** | 0.000 |
| Among individuals  within populations | 64 | 138.544 | -0.340 | -12.69 | *F_IS_* = -0.136 | 1.000 |
| Within individuals | 68 | 193.500 | 2.846 | 106.06 | *F_IT_* = -0.061 | 1.000 |
| Total | 135 | 354.794 |  |  |  |  |

d.f., degrees of freedom.

**P* < 0.05; ***P* < 0.01; ****P* < 0.001.
