# Supplementary figures and images for: Banat donkey, a neglected donkey breed from the central Balkans (Serbia)
Source: PeerJ. 2020 Mar 3;8:e8598. doi: 10.7717/peerj.8598 (PMC7059758; doi:10.7717/peerj.8598)

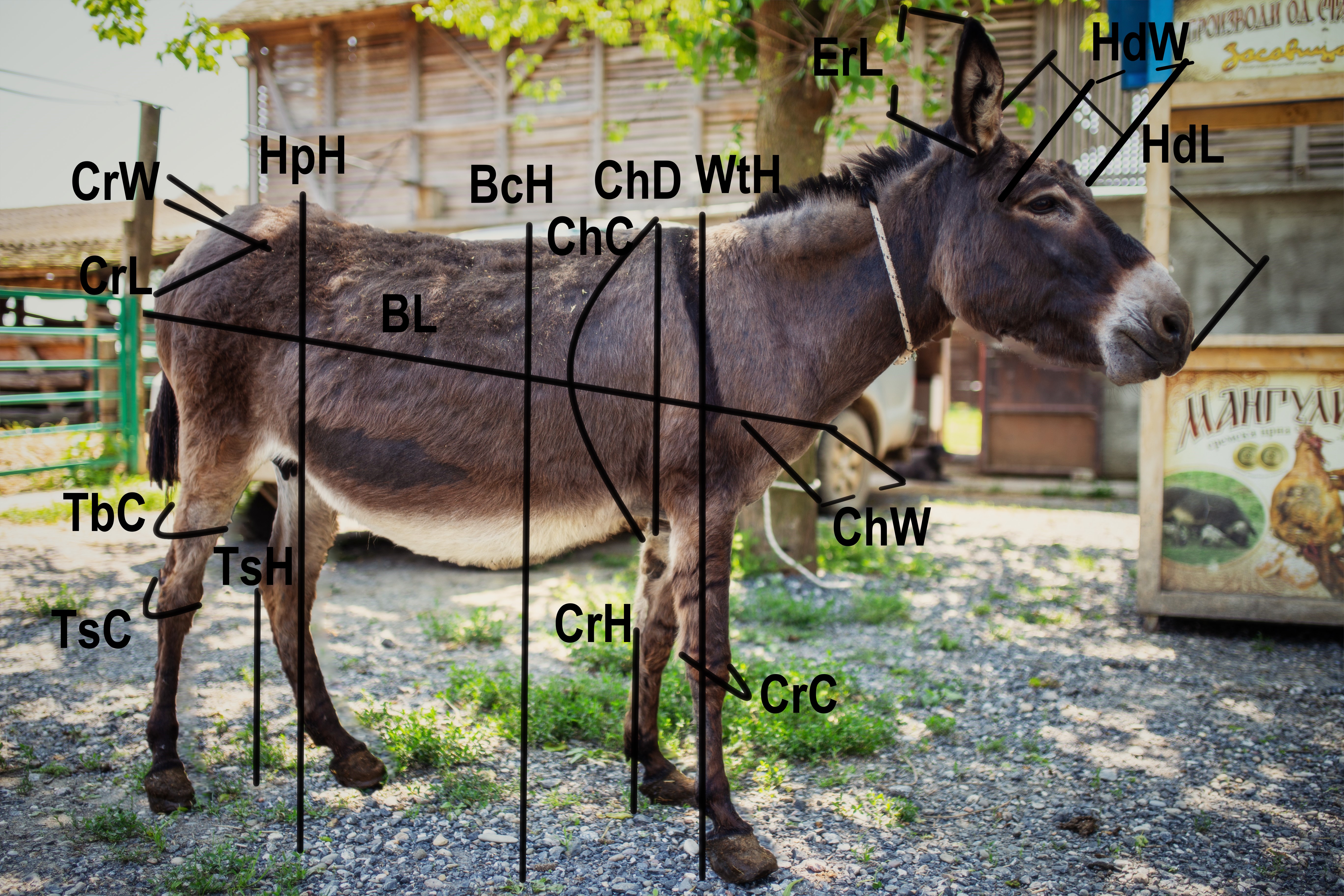

Supplement: Figure S1 — Abbreviations of measured morphological traits: BcH, Back height (cm); BL, Body length (cm); bw, Body weight (kg); CrC, Carpal circumference (cm); CrH, Carpal height (cm); ChC, Chest circumference (cm); ChD, Chest depth (cm); ChW, Chest width (cm); CrL, Croup length (cm); CrW, Croup width (cm); ErL, Ear length (cm); HdL, Head length (cm); HdW, Head width (cm); HpH, Hip height (cm); TsC, Tarsal circumference (cm); TsH, Tarsal height (cm); TbC, Tibia circumference (cm); WtH, Wither height (cm). [file peerj-08-8598-s003.jpg]

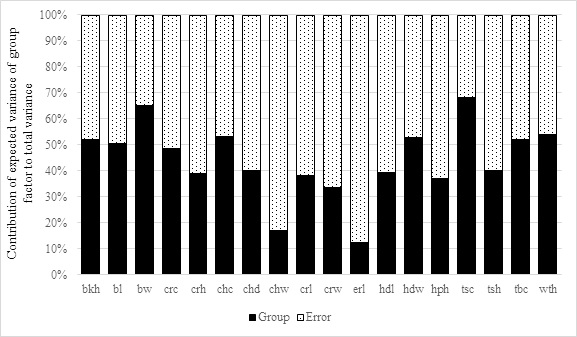

Supplement: Figure S2 — Abbreviations of measured morphological traits: bkh, Back height (cm); bl, Body length (cm); bw, Body weight (kg); crc, Carpal circumference (cm); crh, Carpal height (cm); chc, Chest circumference (cm); chd, Chest depth (cm); chw, Chest width (cm); crl, Croup length (cm); crw, Croup width (cm); erl, Ear length (cm); hdl, Head length (cm); hdw, Head width (cm); hph, Hip height (cm); tsc, Tarsal circumference (cm); tsh, Tarsal height (cm); tbc, Tibia circumference (cm), wth, Wither height (cm). Groups are Banat donkey (BanD), potential hybrids (HY), and two sub-populations of the Balkan donkey, BalkD-BGP and BalkD-RGP. [file peerj-08-8598-s004.jpg]

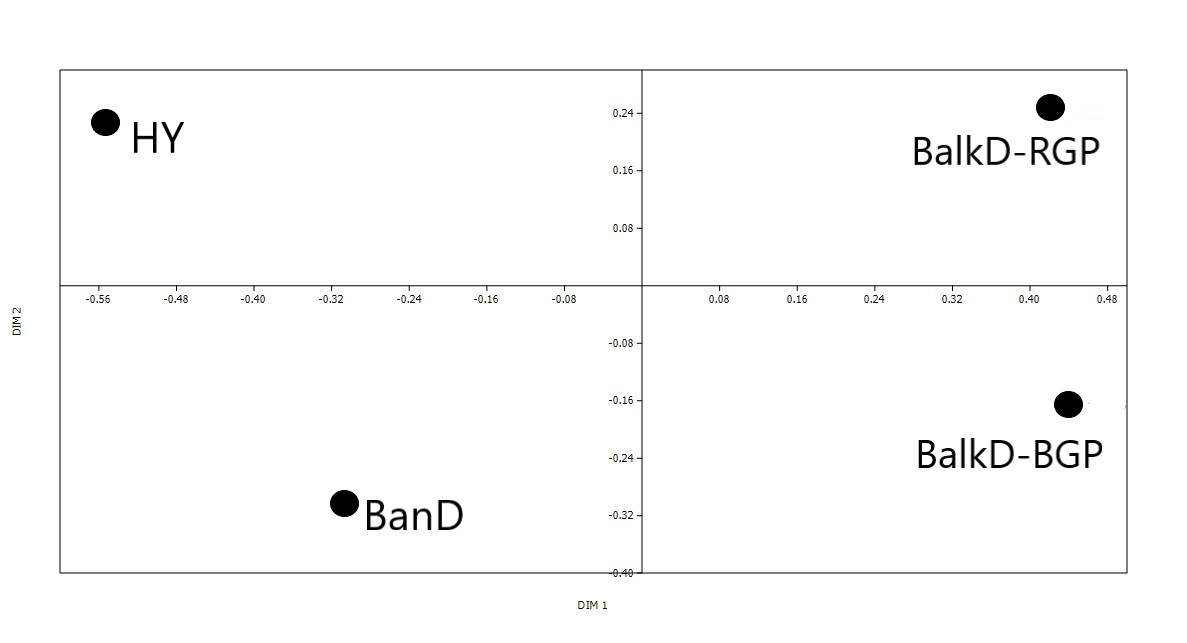

Supplement: Figure S3 [file peerj-08-8598-s005.jpg]

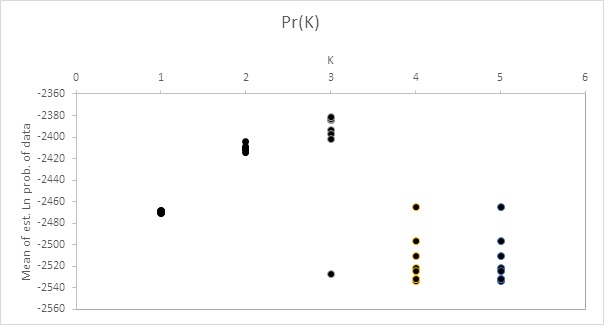

Supplement: Figure S4 [file peerj-08-8598-s006.jpg]
